# Invasive ductal carcinoma of the breast with gallbladder metastasis: a rare case report

**DOI:** 10.1186/s12957-025-04013-8

**Published:** 2025-10-13

**Authors:** Kendall Vignaroli, Kevin Perez, Michelle Lee, Sharmila Raju, Alex Nguyen, Aldin Malkoc, Ella Martinetto, Ruben Burbank, Ahmad Ibrahim, Ellen Ko, Judi Anne B. Ramiscal

**Affiliations:** 1 Department of General Surgery, Arrowhead Regional Medical Center, 400 N Pepper Ave, Colton, CA 92324 USA; 2 Department of Surgical Oncology, Arrowhead Regional Medical Center, Colton, CA USA; 3 School of Medicine, California University of Science and Medicine, Colton, CA USA; 4 Department of Laboratory Medicine, Arrowhead Regional Medical Center, Colton, CA USA

**Keywords:** Breast neoplasm, Invasive ductal carcinoma, Breast conserving surgery, Gallbladder metastasis, Rare metastasis site, Visceral metastasis

## Abstract

**Background:**

Invasive ductal carcinoma of the breast most commonly metastasizes to bone, lung, liver, and central nervous system. Breast cancer metastasis to the gallbladder is exceptionally rare, especially when it is secondary to breast cancer of ductal origin.

**Case presentation:**

We present the case of a pre-menopausal 43-year-old female with a history of major depressive disorder and no prior mammograms who was diagnosed with ER+/PR+/HER2+ invasive ductal carcinoma of the right breast. She developed late metastasis to the gallbladder, liver, lung and bone detected four years after breast conserving surgery with delayed neoadjuvant chemotherapy, adjuvant radiation, incomplete adjuvant biologic and hormone therapy, and lack of surveillance. The patient died three years and ten months after her lumpectomy.

**Conclusions:**

Though rare, adequate suspicion should be maintained when evaluating patients with a history of breast cancer who present with symptoms of cholecystitis or biliary colic in order to promptly identify breast cancer metastasis to the gallbladder, as well as to more common metastatic sites.

## Background

Invasive ductal carcinoma (IDC) of the breast is a malignancy which originates in the epithelial cells of milk ducts and infiltrates the ductal walls into the fatty portion of the breast [1]. IDC is the most common type of breast cancer, accounting for 80%−85% of all breast cancer diagnoses in women [1–4]. The approach to treatment of IDC can involve breast conservation surgery with post-operative radiation or mastectomy, and iterations of this treatment vary based on shared decision making with the patient. The addition of chemotherapy or hormonal therapy depends on additional factors including stage, nodal status, and receptor status [5]. IDC has been shown to have distant metastasis rates between 3%−14%[4, 6, 7], and these sites of metastasis occur most commonly in bone, lung, liver, and central nervous system [3, 7]. Breast cancer metastasis to the gallbladder is exceptionally rare and seen most commonly in patients with invasive lobular carcinoma (ILC) [8–11] or mixed lobular and ductal origin [12, 13]. Breast cancer metastasis to the gallbladder is even more rarely reported from breast cancer with ductal origin [14, 15].

In this case, we describe a 43-year-old female with major depressive disorder who underwent delayed neoadjuvant chemotherapy and breast conserving surgery for estrogen receptor positive (ER+), progesterone receptor positive (PR+), and human epidermal growth factor − 2 positive (HER2+) invasive ductal carcinoma with adjuvant radiation and an incomplete course of adjuvant biologic and hormone therapy. This patient did not complete her recommended surveillance, and subsequently presented with metastasis within the gallbladder, lungs, liver, and bones more than three years after her initial lumpectomy.

## Case presentation

A 43-year-old female with a medical history of major depressive disorder and no prior mammograms presented in August of 2020 with a one-year history of skin dimpling over the right superomedial breast and a two-month history of a palpable mass in that same area. At this time the patient was pre-menopausal with regular menses, and had no known family history of breast cancer or ovarian cancer. She underwent diagnostic mammography which revealed a hypoechoic and mildly heterogeneous irregular mass at the superomedial aspect of the right breast, ten centimeters from the nipple measuring 3.6 x. 2.4 × 3.5 cm, and a 1.6 × 0.9 × 2.1 cm lymph node in the right axilla which was noted to be normal appearing. A core needle biopsy of the right breast mass showed infiltrating ductal carcinoma, receptors ER+/PR+/HER2+. The patient was determined to have Stage IB disease (cT2, cN0, cM0.) Neoadjuvant chemotherapy with docetaxel, carboplatin, trastuzumab, and pertuzumab (TCHP) was scheduled for six cycles to be given three weeks apart. Her first cycle was delayed until ten weeks after tissue biopsy due to a lack of insurance, and her fourth cycle was delayed three months due to a lapse in insurance. The patient underwent right lumpectomy ten months after tissue diagnosis, where the breast mass was resected and five sentinel lymph nodes were removed. The final pathology revealed residual invasive ductal carcinoma measuring 1.3 cm with surgical margins free of tumor and no lymphovascular invasion; all five sentinel axillary nodes were negative for metastasis. The patient was then scheduled to begin trastuzumab for 14 cycles with concomitant adjuvant whole breast radiation, followed by goserelin and tamoxifen. She completed adjuvant whole breast radiation three months post lumpectomy. Four months post lumpectomy, surveillance mammogram showed left inferomedial quadrant low-density mass in the middle depth, however this was not reproduced on ultrasound, which demonstrated only fatty breast tissue. Five months post lumpectomy, the joint decision was made to halt all interventions including trastuzumab, tamoxifen, and goserelin due to suicidal ideation. After this decision was made, the patient did not present for her subsequent appointments and did not complete her next surveillance mammogram planned for ten months post lumpectomy.

**Fig. 1:**
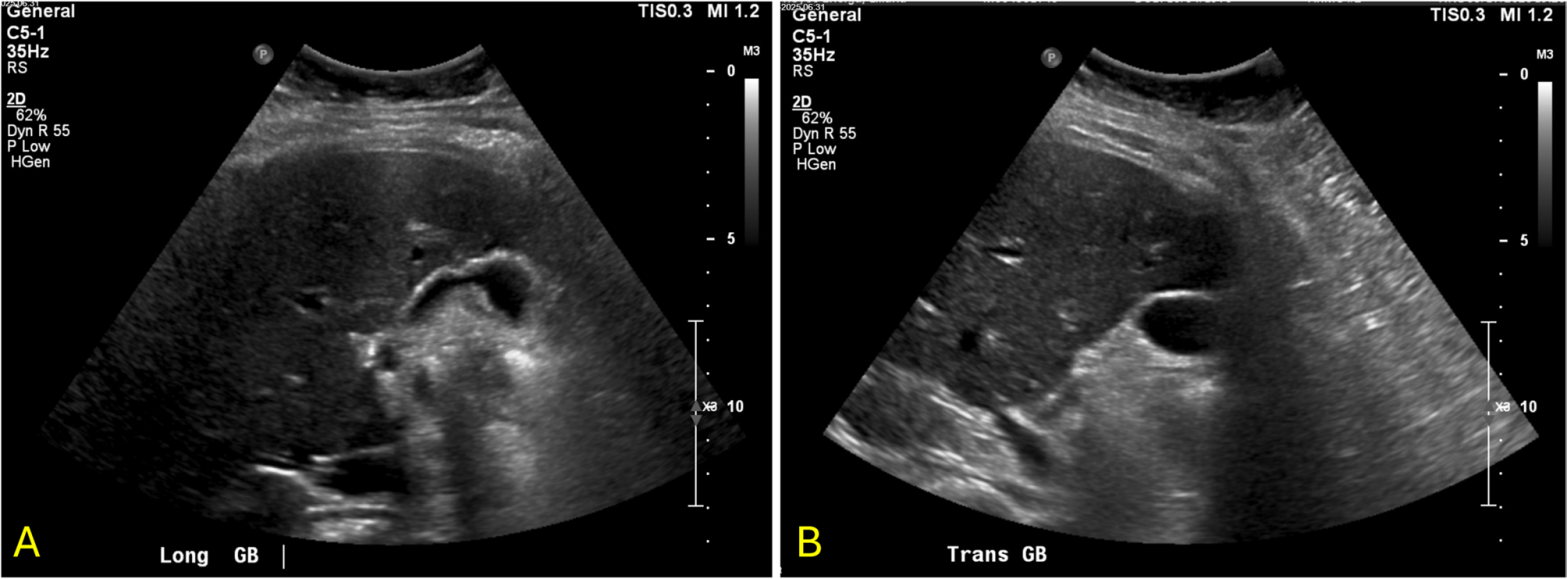
Ultrasound of the gallbladder and surrounding liver. **A** Longitudinal view and **(B)** transverse view demonstrating gallbladder without echogenic shadowing or wall thickening, and homogenous liver parenchyma with no clearly visible liver mass

**Fig. 2:**
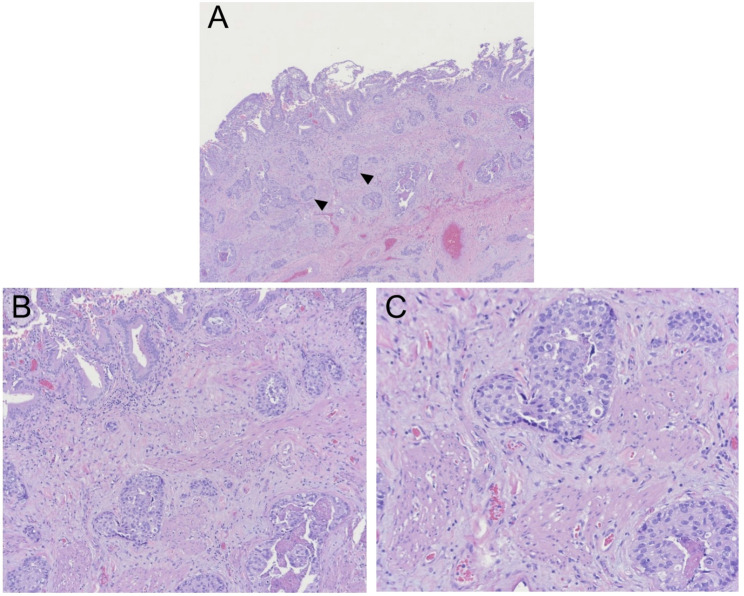
Gallbladder specimen showing evidence of cholesterolosis in the mucosa and nests of poorly differentiated invasive carcinoma with necrosis in the submucosa. **A** Hematoxylin-eosin, original magnification x4, black arrows indicate nests of tumor cells. **B** Higher magnification x10 and **(C)** higher magnification x20 of image showing nests of malignant cells with high nuclear to cytoplastic (N/C) ratios, hyperchromatic nuclei, and prominent nucleoli. Lymphovascular invasion of carcinoma also visualized

**Fig. 3:**
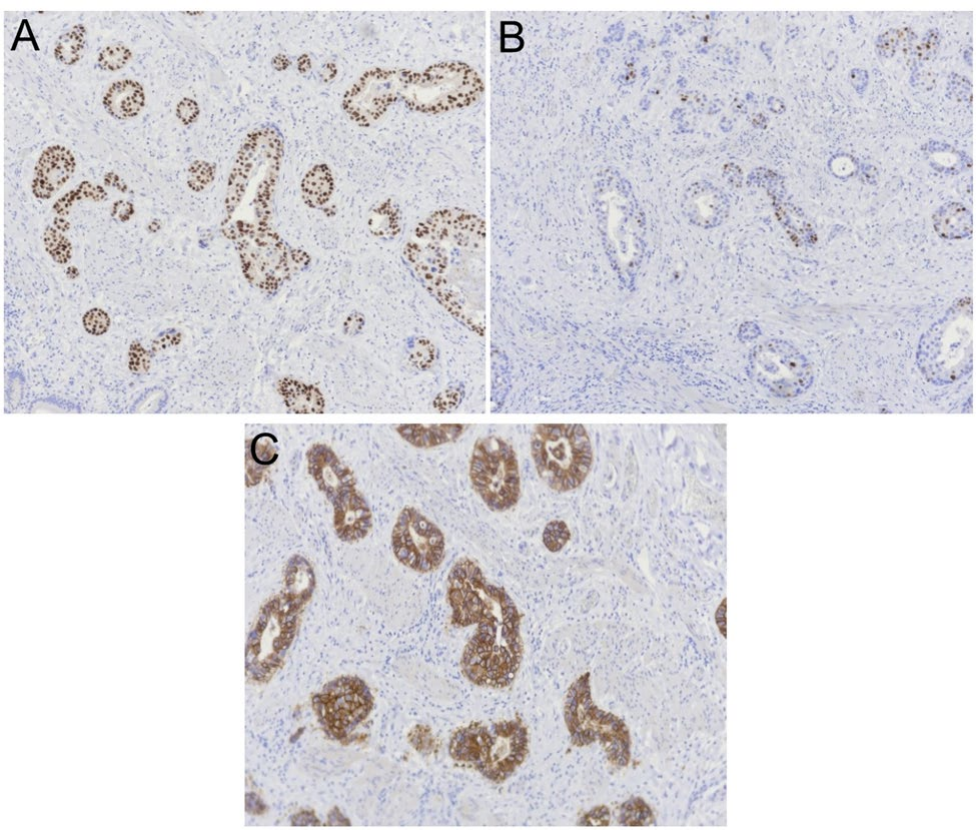
**A** Immunostaining for estrogen receptor (ER) shows greater than 90% of tumor cells demonstrating strong nuclear staining indicating a positive result. **B** Immunostaining for progesterone receptor (PR) shows approximately 50–60% of tumor cells with moderate staining indicating a positive result. **C** Immunostaining for human epidermal growth factor − 2 (HER2) shows circumferential membrane staining that is complete, intense, and in > 10% of tumor cells indicating an IHC (score 3+)

**Fig. 4:**
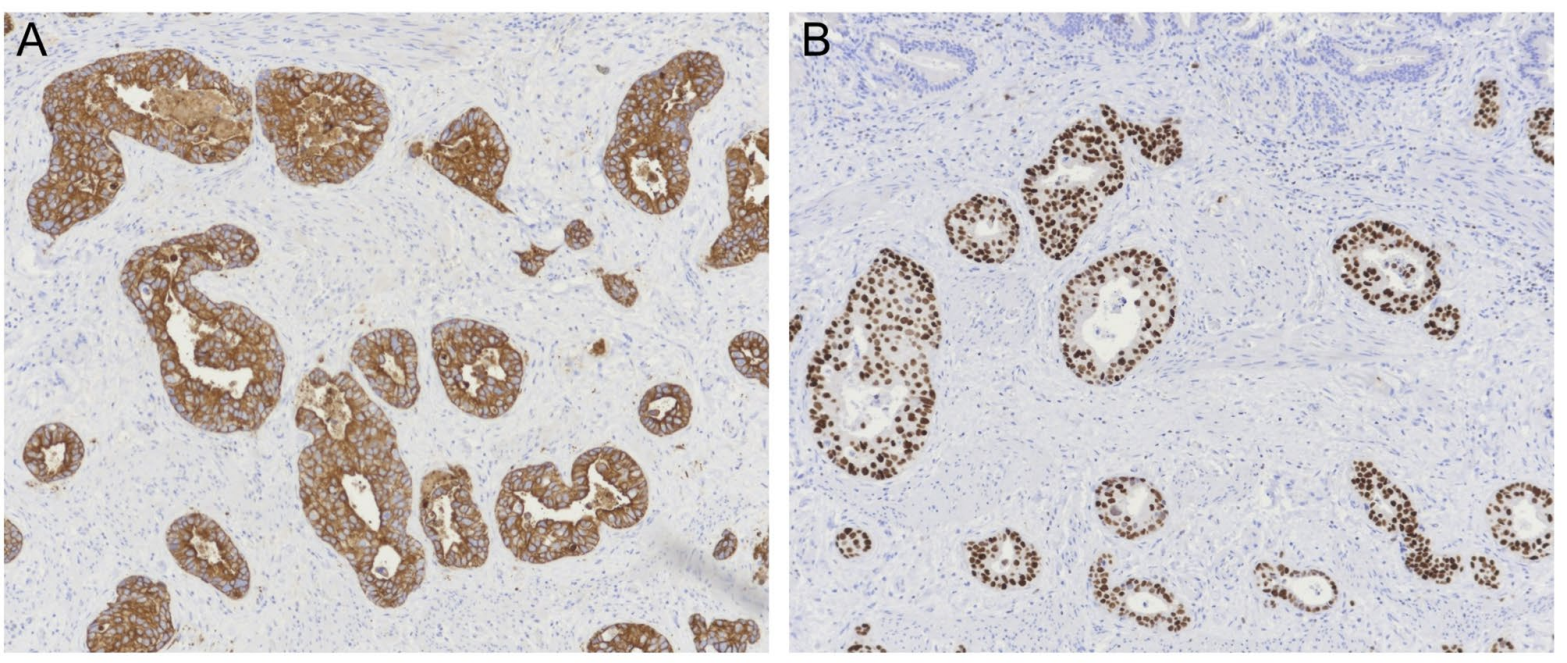
**A** Immunostaining for cytokeratin 7 (CK7) shows strong, diffuse staining in > 10% of tumor cells indicating a positive result. **B** Immunostaining for GATA binding protein 3 (GATA3) shows greater than 90% of cells demonstrating strong nuclear staining indicating a positive result

The patient subsequently developed symptoms of nausea, vomiting, decreased appetite, fatigue, and productive cough three years and eight months post lumpectomy. One month later, she presented to a different hospital with symptoms of right upper quadrant abdominal pain which began after eating a large meal. She had no symptoms of vomiting, diarrhea, fevers or chills at presentation, however she did report a two-month history of dry cough exacerbated by exertion. On exam the patient was tender to palpation over the epigastric and right upper abdomen with a positive Murphy’s sign. Her labs were significant for a total bilirubin of 1.4 mg/dL (reference range 0.0-1.2 mg/dL), alkaline phosphatase of 211 U/L (35–125 U/L), alanine aminotransferase (ALT) of 126 U/L (5–40 U/L), and aspartate aminotransferase (AST) of 135 U/L (5–40 U/L). She had no leukocytosis and her vitals were within normal limits. Ultrasound of the gallbladder completed by a technician demonstrated echogenic densities without shadowing, questionable gallbladder sludge, and an unremarkable gallbladder wall (Fig. 1); the radiologist report also noted no appreciation of ascites or liver masses. The patient was diagnosed with early acute cholecystitis and underwent laparoscopic cholecystectomy the following day where her gallbladder was noted to have a markedly inflamed and friable serosa. Other intra-operative findings noted a firm, nodular liver as well as adhesions connecting the gallbladder to omentum and duodenum; otherwise, there was no mention of peritoneal implants seen intraoperatively. The patient was discharged home from the hospital two days post cholecystectomy. Pathology reported a focally thickened gallbladder wall showing infiltration by poorly differentiated invasive carcinoma with necrosis (Fig. 2) compatible with metastatic carcinoma of the breast origin, likely invasive mammary ductal carcinoma. Immunohistochemistry stains were positive for CK7, CK19, CAM5.2, GATA3, ER, PR, HER2, Ki-67 (> 40%) (Figs. 3 and 4); stains were negative for TTF1, CDX2, CD56, ChromograninA, and Synaptophysin. The patient was notified of her pathology at a postoperative clinic visit ten days after surgery and was referred to surgical and medical oncology. Prior to follow-up with outpatient oncology, she was admitted to the hospital 18 days after cholecystectomy with complaints of epigastric pain, vomiting, and shortness of breath. Subsequent computed tomography (CT) scan of the chest, abdomen, and pelvis showed a poorly defined lesion within the superior-medial aspect of the right breast, and findings concerning for pelvic ascites, liver metastasis, bilateral lung metastasis, and bony metastasis involving the right clavicle, left ribs, and sternum with bony destruction. Inpatient medical oncology was consulted, who recommended initiation of goserelin injections every 28 days as she could not tolerate additional therapy at that time. The patient underwent thoracentesis on hospital day three, with pleural fluid cytology showing malignant effusion and immunohistochemistry pattern consistent with metastatic mammary carcinoma. The patient’s liver enzymes began to rise significantly and a magnetic resonance cholangiopancreatography (MRCP) was recommended, however the patient refused imaging. She was started on trastuzumab and pertuzumab on hospital day nine, however that evening she had increased oxygen requirements and worsening mentation necessitating upgrade to the intensive care unit. The patient was then placed on comfort care and time of death was called on hospital day ten, three years and ten months after her lumpectomy (Table 1).

**Table 1:** Clinical timeline

August 2020	Patient presents with right breast skin dimpling; biopsy shows infiltrating ductal carcinoma
November 4, 2020	C1 of TCHP completed (delayed due to lapse in insurance)
November 25, 2020	C2 completed
December 16, 2020	C3 completed
March 18, 2021	C4 completed (delayed for 3 months due to loss of insurance)
April 8, 2021	C5 completed
April 29, 2021	C6 completed
June 15, 2021	Right lumpectomy and sentinel lymph node biopsy completed
July 12, 2021	Trastuzumab initiated
September 13, 2021	Goserelin initiated
September 16, 2021	Whole breast radiation completed
September 23, 2021	Tamoxifen initiated
October 1, 2021	Baseline bone density scan shows osteopenia
October 19, 2021	Mammogram shows a left lower inner quadrant low-density mass in the middle depth, however this was not reproduced on ultrasound
November 15, 2021	Trastuzumab, tamoxifen, and goserelin halted due to suicidal ideation
April 2022	Patient foregoes her scheduled 6 month surveillance imaging, not completed
March 18, 2025	Laparoscopic cholecystectomy completed
March 28, 2025	Patient is notified of her gallbladder metastasis diagnosis in General Surgery clinic. Referrals are placed to medical oncology, surgical oncology, and tumor board
April 5, 2025	Patient presents to emergency department with shortness of breath and epigastric pain; bone, lung, and liver metastasis discovered on imaging
April 14, 2025	Herceptin and Perjeta initiated
April 15, 2025	Patient dies

## Discussion

IDC of the breast most commonly metastasizes to bone, lung, liver, and central nervous system [3, 7]and breast cancer metastasis to the gallbladder is exceptionally rare. In 2019 Di Micco et al. completed a literature review of 428 papers reporting rare sites of breast cancer metastasis, and found only eight reports of breast cancer metastasis to the gallbladder [16]. Breast cancer metastasis to the gallbladder is even more rare when the primary breast tumor involves invasive ductal carcinoma [9, 14, 15]. Our patient was diagnosed with invasive ductal carcinoma of the right breast with receptors ER+/PR+/HER2+, underwent delayed neoadjuvant chemotherapy, breast conserving surgery, adjuvant radiation, and an incomplete course of adjuvant biologic therapy and hormone therapy. The patient did not return for recommended surveillance, and subsequently presented more than three years later with metastasis to the gallbladder, liver, lungs, and bones.

Our literature review includes eight papers each describing one patient with breast cancer metastasis to the gallbladder: the majority of which involve primary breast cancer of lobular origin, receptors ER+, PR+, and HER2-, and a discovery of gallbladder metastasis made after cholecystectomy was performed for a diagnosis of biliary colic or cholecystitis (Table 2) [8–15]. Gallbladder metastasis secondary to HER2 + breast cancer as described in our case is rarely reported in literature, however studies have shown that HER2 + status does appear to predispose to the development of visceral metastasis. A multi-center retrospective study conducted over the course of three years in China showed that HER2-enriched and Luminal B cases increase the risk of visceral metastasis by 46% and 23%, with liver metastasis being the most frequently observed metastasis in HER2-enriched patients [17]. Another retrospective study conducted in Turkey over the course of six years found a significantly higher instance of visceral metastasis in the presence of HER2 + receptors[18]. The propensity to develop visceral metastasis in the setting of HER2 + receptors lies in the biology of HER2 over-expression. HER2 facilitates epithelial-mesenchyme transition and matrix metalloproteinases which allow cells to migrate and invade nearby tissues via the extracellular matrix. HER2 signaling pathways also upregulate intravasation and angiogenesis, processes which facilitate cells to enter the bloodstream or lymphatic vessels, disseminate to distant organs, and provide vital nutrition and oxygen to metastatic tumors. Finally, HER2 enhances the PI3K/Akt/mTOR pathway which promotes migration and survival of malignant cells in other metastatic locations [19]. Therefore, the gallbladder metastasis described in this case report involving HER2 + breast cancer was likely secondary to hematogenous spread via systemic circulation. However, the gastrointestinal nature of the metastasis in our case is rare in the setting of breast IDC. Literature shows that ILC of the breast is more likely to metastasize to the gastrointestinal tract, which is attributed to its absence of E-cadherin expression, an intercellular adhesion molecule [20]. Therefore it is thought that ILC spreads in a more diffuse, infiltrative pattern [20] compared to IDC, which more often has lymphatic spread [2].

**Table 2:** Case reports describing breast cancer with metastasis to the gallbladder

Author	Publication year	Patient age (years)	Breast pathology	Breast cancer laterality	Receptors	Oncologic breast resection	Metastasis sites (other than gallbladder)	Documented indication for cholecystectomy	Survival
Molina-Barea[8]	2014	62	Infiltrating lobular carcinoma	Left	ER+/PR-/HER2-	Mastectomy	Right colon	Complicated biliary colic	Expired
Zamkowski[9]	2017	64	Invasive lobular carcinoma	Bilateral	ER+/PR-/HER2-	Not completed	Stomach, pancreas	Biliary colic, acute cholecystitis	Alive
Bezpalko[10]	2015	47	Invasive lobular carcinoma	Right	ER+/PR+/HER2-	Not completed	Bone marrow, endometrium, regional lymph nodes, and peritoneum	Acute cholecystitis	Expired
Abdelilah[11]	2014	45	Invasive lobular carcinoma	Right	ER+/PR+/HER2+	Mastectomy	NR	Acute cholecystitis	NR
Markelov[12]	2011	67	Infiltrative lobular carcinoma, ductal carcinoma in situ	Right	ER+/PR+/HER2-	Modified radical mastectomy	NR	Gallbladder dyskinesia	NR
Zagouri[13]	2007	59	Right: invasive lobular carcinoma Left: invasive ductal carcinoma	Bilateral	Right: ER+/PR+/HER2- Left: ER+/PR+/HER2+	Right: modified radical mastectomy Left: lumpectomy	None	Cholecystitis	Alive
Missori[14]	2020	83	Invasive ductal carcinoma	Left	ER+/PR+/HER2-	“Breast surgery”	Vertebral bone, peritoneum	Biliary sepsis due to acute cholecystitis, choledocholithiasis	Expired
Ebrahim[15]	2015	65	Inflammatory invasive mammary carcinoma	Left	ER+/PR+/HER2-	NR	Multiple pulmonary masses, right hepatic lobe lesion, several thoracic vertebrae osteolytic lesions	Right upper quadrant abdominal pain	NR

*NR* Not recorded

The medical management of metastatic breast cancer is nuanced and dependent on the presence of bone disease and receptor status. For stage IV (M1) disease with the presence of bone disease, the NCCN guidelines recommend the addition of denosumab, zoledronic acids or pamidronate. For stage IV (M1) disease with receptors PR + and HER2+, the guidelines recommend the addition of HER2-targeted systemic therapy or endocrine therapy with or without HER2-targeted therapy until unacceptable toxicity is achieved or “progression of disease” occurs, including disease exacerbation at previously known sites or the occurrence of new sites of metastatic disease [5]. Medical management including systemic and local therapy is the mainstay of treatment for breast cancer with distant metastasis, especially in the case of HER2-positive breast cancer. While surgical intervention for breast cancer metastasis has not traditionally been considered the “standard of care” [21]some literature has emerged supporting surgical metastasectomy in the case of oligometastasis where there are a limited number of metastasis and limited number of distinct metastatic locations [21]. This topic is debated because the available data regarding survival after metastasectomy is contradictory and the survival benefit of metastasectomy varies by metastatic site [21, 22]. Because the benefit of metastasectomy remains unclear, definitive guidelines commenting on the pursuit of metastasectomy in stage IV breast cancer do not yet exist.

The literature reports distant metastasis rates of IDC between 3% and 14% and generally assesses patients longitudinally over the course of at least a decade [4, 6, 7]. During this time of follow-up, guidelines recommend completion of history and physical exams one to four times per year for five years and then annually thereafter, mammography every 12 months beginning six months after breast conserving treatment, as well as work up for any abnormal review of systemic findings [5]. However the metastasis rates reported longitudinally in the literature usually exclude patients who have not completed the full course of recommended adjuvant therapy, and likely exclude or fail to capture patients who do not report for the recommended surveillance follow-up. As such, it is difficult to glean longitudinal data regarding rates of metastasis in patients with IDC who are treated with delayed or incomplete therapy, or in those who do not complete surveillance follow-up. Our case provides valuable insight into the natural course of IDC with suboptimal neoadjuvant and adjuvant systemic therapy and lack of surveillance, and further studies and case reports will need to be published to understand if breast cancer metastasis to rare sites like the gallbladder is more prevalent in cases of breast cancer with delayed neoadjuvant chemotherapy, incomplete adjuvant treatment, or no follow-up surveillance, as well as the incidence of synchronous metastasis to other sites.

Notably, our study describes a significant delay in initiation of neoadjuvant chemotherapy and delay to resection, as well as a lack of surveillance due to lapses and loss of insurance. Lack of resources like access to insurance can significantly delay access to neoadjuvant treatment, thus delaying time to oncologic resection. Studies show that delayed time to resection of breast cancer is associated with increased mortality [23]worse residual cancer burden, and worse recurrence-free survival [24]. Literature also consistently shows that low socioeconomic status is associated with larger tumor size and advanced stage upon presentation of breast cancer, decreased completion of adjuvant radiation and chemotherapy, as well as worse overall survival [25, 26]. Our case highlights the importance of implementation of social programs in promoting equal outcomes and survival for all who present with breast cancer.

One limitation in the understanding of this case’s diagnostic workup and management lies in the constraint inherent to outside record review. Because the patient’s initial diagnosis, workup, and treatment of breast cancer was completed at an outside hospital, this history is limited to that which was collected by acquired outside records. This includes a lack of information regarding whether initial staging workup included a CT scan or bone scan, data to determine the patient’s pathologic response after chemotherapy, unknown histoprognostic factors included in the breast lumpectomy specimen, or the residual cancer burden (RCB) after lumpectomy. There was no clear documentation addressing if trastuzumab emtansine (T-DM1) was considered in the setting of partial pathological response post-neoadjuvant therapy. It would also be useful to understand if medical therapy was already in place treating the patient’s previous diagnosis of major depressive disorder, and why the choice was made to halt all adjuvant therapy in the setting of suicidal ideation instead of first pursuing other treatment avenues including psychiatric support in order to continue anti-HER2 therapy. This is particularly important to consider given the aggressive nature of HER2 + tumors with a partial treatment response. Another limitation in this case was the initial diagnostic workup of the patient’s biliary symptoms and delayed detection of liver metastasis. The large size of liver metastasis eventually detected on CT scan were not detected on initial gallbladder ultrasound. This oversight could have been secondary either to incomplete ultrasound imaging by the technician or an oversight by the reading radiologist. Regardless, there should have been a high suspicion for gallbladder inflammation secondary to liver or biliary metastasis given the patient’s history of HER2 + breast cancer with incomplete adjuvant therapy and lack of surveillance. It is possible that the presence of a specialized imaging protocol or a radiologist with expertise in discerning subtle metastatic disease might have yielded an earlier detection of liver metastasis on the initial ultrasound workup. As such, healthcare systems might consider establishing specialized imaging protocols and the use of radiologists with expertise in oncologic interpretation when obtaining imaging in a patient with a history of breast cancer, regardless of the imaging indication. Because the patient showed no signs of sepsis or evidence of gallstones on ultrasound imaging, additional time could have been spent completing a CT scan for further metastatic workup prior to the completion of cholecystectomy. This highlights the importance of maintaining adequate suspicion of metastasis when evaluating patients with a history of incompletely treated HER2 + breast cancer who present with symptoms of cholecystitis or biliary colic in order to promptly identify breast cancer metastasis to the gallbladder, in addition to metastasis to other more common sites.

## Conclusion

We describe a rare contemporary case of breast cancer metastasis to the gallbladder emerging more than three years post lumpectomy. This case illustrates the natural course of HER2 + invasive ductal carcinoma after delayed neoadjuvant chemotherapy, breast conserving treatment, and incomplete course of adjuvant biologic and hormone therapy with no surveillance. Though rare, adequate suspicion should be maintained when evaluating patients with a history of incompletely treated breast cancer who present with symptoms of cholecystitis or biliary colic in order to promptly identify breast cancer metastasis to the gallbladder, in addition to metastasis to other more common sites

## Data Availability

No datasets were generated or analysed during the current study.

## Abbreviations

IDC: Invasive ductal carcinoma
ILC: Invasive lobular carcinoma
ER+: Estrogen receptor positive
PR+: Progesterone receptor positive
HER2+: Human epidermal growth factor − 2 positive
ALT: Alanine aminotransferase
AST: Aspartate aminotransferase
MRCP: magnetic resonance cholangiopancreatography

## References

[1] Sharma GN, Dave R, Sanadya J, Sharma P, Sharma KK. Various types and management of breast cancer: an overview. J Adv Pharm Technol Res. 2010;1(2):109–26. 22247839 PMC3255438

[2] Molland JG, Donnellan M, Janu NC, Carmalt HL, Kennedy CW, Gillett DJ. Infiltrating lobular carcinoma–a comparison of diagnosis, management and outcome with infiltrating duct carcinoma. Breast. 2004;13(5):389–96. 10.1016/j.breast.2004.03.004. 15454194 10.1016/j.breast.2004.03.004

[3] Duraker N, Hot S, Akan A, Nayır PÖ. A comparison of the clinicopathological features, metastasis sites and survival outcomes of invasive lobular, invasive ductal and mixed invasive ductal and lobular breast carcinoma. Eur J Breast Health. 2020;16(1):22–31. 10.5152/ejbh.2019.5004. 31912010 10.5152/ejbh.2019.5004 PMC6939713

[4] Chen BF, Tsai YF, Lien PJ, Lin YS, Feng CJ, Chen YJ, Cheng HF, Tseng LM, Huang CC. Clinical characteristics and treatment outcomes of invasive ductal and lobular carcinoma: analyses of 54,832 Taiwan cancer registry index cases. Breast Cancer Res Treat. 2023;201(3):547–60. 10.1007/s10549-023-07044-5. 37470893 10.1007/s10549-023-07044-5

[5] National Comprehensive Cancer Network (NCCN). *NCCN Clinical Practice Guidelines in Oncology: Breast Cancer. Version 4.2024* [Internet]. Plymouth Meeting (PA): NCCN; 2024 [cited 2025 June 10]. Available from: https://www.nccn.org/guidelines/guidelines-detail?category=1&id=1419

[6] Park JS, Choi DH, Huh SJ, Park W, Kim YI, Nam SJ, Lee JE, Kil WH. Comparison of clinicopathological features and treatment results between invasive lobular carcinoma and ductal carcinoma of the breast. J Breast Cancer. 2015;18(3):285–90. 10.4048/jbc.2015.18.3.285. 26472980 10.4048/jbc.2015.18.3.285 PMC4600694

[7] Cao AY, Huang L, Wu J, Lu JS, Liu GY, Shen ZZ, Shao ZM, Di GH. Tumor characteristics and the clinical outcome of invasive lobular carcinoma compared to infiltrating ductal carcinoma in a Chinese population. World J Surg Oncol. 2012;10:152. 10.1186/1477-7819-10-152. 22805492 10.1186/1477-7819-10-152 PMC3502188

[8] Molina-Barea R, Rios-Peregrina RM, Slim M, Calandre EP, Hernández-García MD, Jimenez-Rios JA. Lobular breast cancer metastasis to the colon, the appendix and the gallbladder. Breast Care. 2014;9(6):428–30. 10.1159/000368430. 25759626 10.1159/000368430 PMC4317682

[9] Zamkowski M, Kąkol M, Makarewicz W, Ropel J, Bobowicz M. Patient with metastatic breast cancer presenting as acute cholecystitis with one-year survival on hormonotherapy. Pol Przegl Chir. 2017;89(4):46–9. 10.5604/01.3001.0010.4063. 28905808 10.5604/01.3001.0010.4063

[10] Bezpalko K, Mohamed MA, Mercer L, McCann M, Elghawy K, Wilson K. Concomitant endometrial and gallbladder metastasis in advanced multiple metastatic invasive lobular carcinoma of the breast: A rare case report. Int J Surg Case Rep. 2015;14:141–5. 10.1016/j.ijscr.2015.07.036. 26275738 10.1016/j.ijscr.2015.07.036 PMC4573862

[11] Abdelilah B, Mohamed O, Yamoul R, Elkhiyat I, Al Bouzidi A, Alkandry S, et al. Acute cholecystitis as a rare presentation of metastatic breast carcinoma of the gallbladder: a case report and review of the literature. Pan Afr Med J. 2014;17:216. 10.11604/pamj.2014.17.216.3911. 25237413 10.11604/pamj.2014.17.216.3911 PMC4163176

[12] Markelov A, Taheri H, Vunnamadala K, Ibrahim G. Biliary dyskinesia as a rare presentation of metastatic breast carcinoma of the gallbladder: a case report. Case Rep Pathol. 2011;2011:806570. 10.1155/2011/806570. Epub 2011 Sep 21. PMID: 22937393; PMCID: PMC3420450. 22937393 10.1155/2011/806570 PMC3420450

[13] Zagouri F, Sergentanis TN, Koulocheri D, Nonni A, Bousiotou A, Domeyer P, Michalopoulos NV, Dardamanis D, Konstadoulakis MM, Zografos GC. Bilateral synchronous breast carcinomas followed by a metastasis to the gallbladder: a case report. World J Surg Oncol. 2007;5:101. 10.1186/1477-7819-5-101. 17848197 10.1186/1477-7819-5-101 PMC2075501

[14] Missori G, Serra F, Prestigiacomo G, Ricciardolo AA, Brugioni L, Gelmini R. Case report: metastatic breast cancer to the gallbladder. F1000Res. 2020;9:343. 10.12688/f1000research.23469.1. 33204409 10.12688/f1000research.23469.1 PMC7610173

[15] Ebrahim H, Graham D, Rice D, Ribadeneyra M, Thorner K, Shipley W, Wehmueller M. Inflammatory metastatic breast cancer with gallbladder metastasis: an incidental finding. J Community Support Oncol. 2015;13(7):256–9. 10.12788/jcso.0154. 26270542 10.12788/jcso.0154

[16] Di Micco R, Santurro L, Gasparri ML, Zuber V, Fiacco E, Gazzetta G, Smart CE, Valentini A, Gentilini OD. Rare sites of breast cancer metastasis: a review. Transl Cancer Res. 2019;8(Suppl 5):S518–52. 10.21037/tcr.2019.07.24. 35117130 10.21037/tcr.2019.07.24 PMC8797987

[17] Fan JH, Zhang S, Yang H, Yi ZB, Ouyang QC, Yan M, et al. Molecular subtypes predict the preferential site of distant metastasis in advanced breast cancer: a nationwide retrospective study. Front Oncol. 2023;13:978985. 10.3389/fonc.2023.978985. 36761968 10.3389/fonc.2023.978985 PMC9905808

[18] Özdemir Akdur P, Çiledağ N. Review of the relationship between tumor receptor subtypes and preference for visceral and/or serosal metastasis in breast cancer patients. Med (Baltim). 2023;102(43):e35798. 10.1097/MD.0000000000035798. PMID: 37904368; PMCID: PMC10615421. 10.1097/MD.0000000000035798 PMC10615421 37904368

[19] Cheng X. A comprehensive review of HER2 in cancer biology and therapeutics. Genes (Basel). 2024;15(7):903. 10.3390/genes15070903. 39062682 10.3390/genes15070903 PMC11275319

[20] Kioleoglou Z, Georgaki E, Koufopoulos N, Kostek O, Volakakis N, Dimitriadou A, et al. Gastrointestinal metastases from lobular breast carcinoma: a literature review. Cureus. 2024;16(7):e65852. 10.7759/cureus.65852. (**PMID: 39219935; PMCID: PMC11364151**). 39219935 10.7759/cureus.65852 PMC11364151

[21] Ueno T. Surgical management of metastatic breast cancer: a mini review. Front Oncol. 2022;12:910544. 10.3389/fonc.2022.910544. 35600412 10.3389/fonc.2022.910544 PMC9114738

[22] Alghamdi MAA, Mahmood Esam S. Role of surgery in metastatic breast cancer: insights from a narrative review. Breast Cancer (Dove Med Press). 2023;15:349–58. 37192867 10.2147/BCTT.S405864 PMC10182804

[23] Hölzel D, Schlesinger-Raab A, Schubert-Fritschle G, Halfter K. Prolonged time to breast cancer surgery and the risk of metastasis: an explorative simulation analysis using epidemiological data from Germany and the USA. Breast Cancer Res Treat. 2025;211(1):151–60. 10.1007/s10549-025-07630-9. 39961969 10.1007/s10549-025-07630-9 PMC11953083

[24] Sutton TL, Schlitt A, Gardiner SK, Johnson N, Garreau JR. Time to surgery following neoadjuvant chemotherapy for breast cancer impacts residual cancer burden, recurrence, and survival. J Surg Oncol. 2020;122(8):1761–9. 10.1002/jso.26216. 33125715 10.1002/jso.26216

[25] Silber JH, Rosenbaum PR, Ross RN, Reiter JG, Niknam BA, Hill AS, et al. Disparities in breast cancer survival by socioeconomic status despite Medicare and Medicaid insurance. Milbank Q. 2018;96(4):706–54. 10.1111/1468-0009.12355. 30537364 10.1111/1468-0009.12355 PMC6287075

[26] Dreyer MS, Nattinger AB, McGinley EL, Pezzin LE. Socioeconomic status and breast cancer treatment. Breast Cancer Res Treat. 2018;167(1):1–8. 10.1007/s10549-017-4490-3. 28884392 10.1007/s10549-017-4490-3 PMC5790605

